# Dengue Infection in Children in Ratchaburi, Thailand: A Cohort Study. I. Epidemiology of Symptomatic Acute Dengue Infection in Children, 2006–2009

**DOI:** 10.1371/journal.pntd.0001732

**Published:** 2012-07-31

**Authors:** Arunee Sabchareon, Chukiat Sirivichayakul, Kriengsak Limkittikul, Pornthep Chanthavanich, Saravudh Suvannadabba, Vithaya Jiwariyavej, Wut Dulyachai, Krisana Pengsaa, Harold S. Margolis, G. William Letson

**Affiliations:** 1 Department of Tropical Pediatrics, Faculty of Tropical Medicine, Mahidol University, Bangkok, Thailand; 2 Ministry of Public Health, Nonthaburi, Thailand; 3 Ratchaburi Hospital, Ministry of Public Health, Ratchaburi, Thailand; 4 Pediatric Dengue Vaccine Initiative, International Vaccine Institute, Seoul, Korea; Pediatric Dengue Vaccine Initiative, United States of America

## Abstract

**Background:**

There is an urgent need to field test dengue vaccines to determine their role in the control of the disease. Our aims were to study dengue epidemiology and prepare the site for a dengue vaccine efficacy trial.

**Methods and Findings:**

We performed a prospective cohort study of children in primary schools in central Thailand from 2006 through 2009. We assessed the epidemiology of dengue by active fever surveillance for acute febrile illness as detected by school absenteeism and telephone contact of parents, and dengue diagnostic testing. Dengue accounted for 394 (6.74%) of the 5,842 febrile cases identified in 2882, 3104, 2717 and 2312 student person-years over the four years, respectively. Dengue incidence was 1.77% in 2006, 3.58% in 2007, 5.74% in 2008 and 3.29% in 2009. Mean dengue incidence over the 4 years was 3.6%. Dengue virus (DENV) types were determined in 333 (84.5%) of positive specimens; DENV serotype 1 (DENV-1) was the most common (43%), followed by DENV-2 (29%), DENV-3 (20%) and DENV-4 (8%). Disease severity ranged from dengue hemorrhagic fever (DHF) in 42 (10.5%) cases, dengue fever (DF) in 142 (35.5%) cases and undifferentiated fever (UF) in 210 (52.5%) cases. All four DENV serotypes were involved in all disease severity. A majority of cases had secondary DENV infection, 95% in DHF, 88.7% in DF and 81.9% in UF. Two DHF (0.5%) cases had primary DENV-3 infection.

**Conclusion:**

The results illustrate the high incidence of dengue with all four DENV serotypes in primary school children, with approximately 50% of disease manifesting as mild clinical symptoms of UF, not meeting the 1997 WHO criteria for dengue. Severe disease (DHF) occurred in one tenth of cases. Data of this type are required for clinical trials to evaluate the efficacy of dengue vaccines in large scale clinical trials.

## Author Summary

There is an urgent need to field test dengue vaccine. Efficacy trials need to be conducted in study sites with sufficiently high dengue incidence to make a robust estimate of vaccine efficacy and where all dengue virustypes circulate frequently. In this paper, we report on dengue disease surveillance on approximately 3000 primary-school children in seven schools in Muang district of Ratchaburi province, central Thailand, from 2006 through 2009. We report on the characteristics of children in this cohort who fell ill with laboratory confirmed dengue disease. The study showed that approximately four percent of the children had laboratory confirmed dengue per year. All four dengue virus types were found to be the causes of illness in children in all seven schools. This study has shown Muang district of Ratchaburi province to be suitable for dengue vaccine testing and the site has been selected for the world’s first dengue vaccine safety and efficacy study, being conducted from 2009–2014 in children aged 4–11 years.

## Introduction

Dengue virus (DENV) infection with any one of the four virus serotypes (DENV-1 to -4), and 4) can produce a spectrum of outcomes, ranging from asymptomatic infection to mild undifferentiated fever (UF), classic dengue fever (DF) and the most severe form of illness, dengue hemorrhagic fever (DHF) [1]. Dengue is an important cause of morbidity and mortality in tropical and subtropical regions of the world [2]. In Thailand, dengue was first recognized in Bangkok in 1958, and in 1987 the largest epidemic ever recorded occurred with 174,285 cases [3]–[5]. Data from 1974 to 1993 showed that dengue was common in children aged less than 15 years of age and the incidence rates among children hospitalized with dengue have been consistently highest in the 5–9 year age group [6]. Disease has been caused by all four DENV serotypes and has become an intractable public health problem in the country [6], [7]. There is no specific antiviral therapeutic licensed for treatment of dengue and prevention relies on mosquito control. As several promising live-attenuated vaccines candidates are in the later stages of clinical development, there is an urgent need to field test dengue vaccines, which may ultimately control the accelerating spread of dengue worldwide [8], [9]. Population-based, laboratory confirmed background data on the epidemiology of dengue in high risk age-specific populations along with field site operational suitability are critical for clinical dengue vaccine trials [8], [10].

Our aims were to collect accurate dengue incidence data for four transmission years in primary school children in a dengue hyper-endemic area, and to establish infrastructure for potential large scale trials of candidate tetravalent dengue vaccine. In 2005, a pilot epidemiologic study of symptomatic dengue infection in 481 school-children aged 3–10 years was conducted, which led to this study conducted during 2006–2009.

## Methods

### Ethics statement

The study protocol was approved by the Ethical Review Committee for Research in Human Subjects, Ministry of Public Health, Thailand, and the Institutional Review Board, International Vaccine Institute, Seoul Korea.

### Study site

The study was carried out in the sub-district Namuang (downtown) of Muang district of Ratchaburi province, which is located approximately 100 km west of Bangkok, and lies between the Maeklong River on the east and the Thai-Myanmar border on the west. The sub-district has a population of 38,835 (census 2006) and a total area of 8.7 km^2^. The principal medical care facility for the province is Ratchaburi Provincial Hospital (RH), a 855-bed tertiary care facility with 90 pediatric beds and 12 pediatricians on staff. In 2005 the hospital served approximately 1,520 outpatients per day. There were 207, 197 and 214 clinically diagnosed dengue patients, admitted to the pediatric dengue ward in 2003, 2004, and 2005, respectively.

### Study design

**Figure 1:**
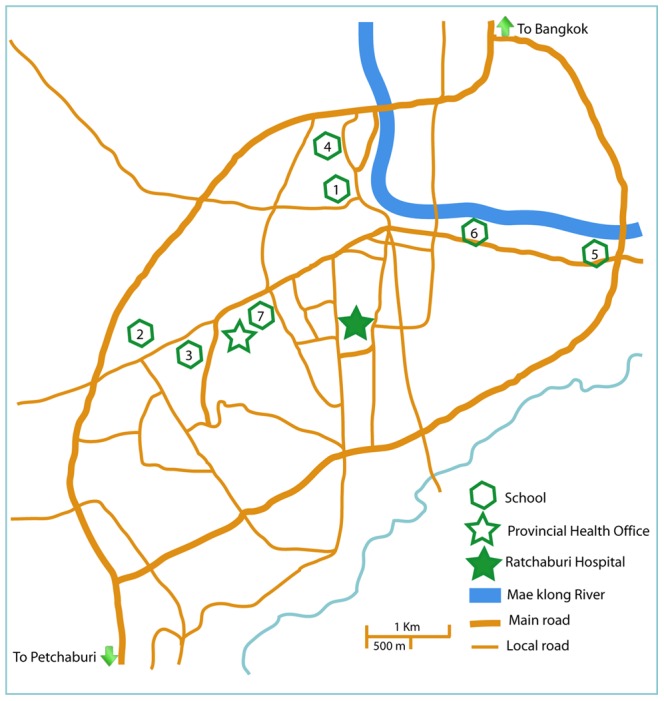
Participating study schools in subdistrict Namuang, district Muang, Ratchaburi province, Thailand, 2006–2009.

This was a prospective cohort study of children attending 7 primary schools. Schools were selected based on their desire to participate in the study, and location within 6 km from RH and the Provincial Health Office (figure 1). Following school-based informational meetings with parents, informed parental consent and signed assent for children >7 years of age were obtained from potential participants. Enrollment criteria were healthy children, no history of chronic illness, ages 3–11 years (grades 1–5) at the time of enrollment, attendance at one of the study schools, and living in a village of Muang district. Exclusion criteria included intent to move outside of the study area within the study period. Children were eligible to remain in the study until graduation from sixth grade. During each January of the study, new children aged 4–5 years were offered the opportunity to enroll to replace children who graduated from the sixth grade.

During the entire four-year study period, active surveillance for school absence and/or children who had a documented fever was conducted by contacting teacher-coordinators daily during school-term and telephoning parents or conducting home visits twice a week during school vacations. School absenteeism was identified each morning by teacher-coordinators at participating schools by comparing names of study participants with reported absences. Participant absenteeism was recorded on a web-based child tracking application at the study field office. Absenteeism was reviewed by the research staff and parents of absent students were contacted by the research staff each afternoon to determine if the child was absent due to a febrile episode.

All parents and teacher-coordinators were provided digital thermometers and were instructed in their use. Parents of a child with a temperature ≥37.5°C were asked to take them to the RH outpatient department (OPD) where there was a special fast track unit with research pediatricians to examine study participants. Children with a fever of ≥38°C or who were considered severely ill were admitted to the inpatient dengue ward (IPD). All illness data were reported at RH on a web- based reporting application.

### Specimen collection

At the OPD or IPD, an acute-phase venous blood sample (S1) was obtained from each febrile study participant and a convalescent-phase venous blood sample (S2) was obtained 7 to 14 days later. S1 and S2 samples were drawn into serum separator tubes, allowed to clot at room temperature for 1–2 h, then stored at 4°C. Serum was separated into aliquots within 24 hours, and stored at –70°C until laboratory testing. Serum samples were transported in dry ice from RH to Bangkok monthly for dengue and Japanese encephalitis (JE) laboratory testing.

### Dengue diagnostic testing

Over the course of the study, diagnostic testing was performed in two laboratories at Mahidol University- Center for Vaccine Development (2006), and at the Faculty of Tropical Medicine, Mahidol (2007–2009) using the same diagnostic algorithm. S1 and S2 were tested for dengue virus specific IgM/IgG by capture enzyme-linked immunosorbent assay (EIA), as described previously [11]. An IgM anti-DENV level ≥40 units was considered indicative of an acute DENV infection. To exclude Japanese encephalitis virus infection and antibody cross-reactivity, specimens were tested concurrently for JE-specific IgM by EIA [11].

S1 samples of the cases with IgM anti-DENV level ≥40 units were further tested for DENV serotype. In 2006, this was performed by mosquito inoculation in *Toxorhynchites splendens* [12] with detection and serotyping by immunofluorrescence. In 2007–9, a modified nested serotype-specific reverse-transcriptase polymerase chain reaction (RT-PCR) [13] was used to serotype DENV.

### Case definitions

#### Dengue

A participant with a febrile illness (oral temperature ≥37.5°C, irrespective of duration) who tested RT-PCR and/or IgM anti-DENV positive on S1 specimen or who seroconverted from IgM anti-DENV negative to positive between S1 and S2 specimens.

#### Primary or secondary DENV infection

Primary DENV infection was defined as an IgM-to- IgG ratio of ≥1.8 by EIA in S1 or S2 specimen. Infection with an IgM-t0-IgG ratio of <1.8 was defined as a secondary DENV infection [11].

#### Dengue hemorrhagic fever (DHF)

The condition was defined as an identified febrile illness with laboratory confirmed DENV infection with evidence of DHF. Clinical criteria and severity grading of DHF were also defined according to the WHO criteria [1].

#### Dengue fever (DF)

The condition was defined as an identified febrile illness with laboratory confirmed DENV infection and meeting the criteria for DF according to the WHO criteria [1].

#### Undifferentiated fever (UF)

This was defined as a person with an acute febrile illness and laboratory confirmed DENV infection who did not meet the WHO clinical criteria for DF or DHF [1].

### Subject identification, data collection, management and quality control

While study participants were tracked by name, school, and home address throughout the study, they were given a unique identification number upon enrollment that was used in the electronic data base for transmittal of epidemiologic, clinical and laboratory data, and for data analyses. Individual identifier information was kept in a secured location separate from data forms. Data were entered within 24 hours after the staff identified a participant as being absent, febrile or at the hospital. Data quality was assured by the study field office and hospital staff managers on a daily basis during weekdays. Data from all sources were automatically transferred through a web-based application to a database located at the Data Management Unit (DMU) of the Faculty of Tropical Medicine, Mahidol University, in Bangkok which uses the Mahidol University Information Technology Department Data Procedure SOP. The DMU monitored inconsistency of data entry daily.

### Statistical analyses

On a monthly basis, the entire dataset for the study children was exported into SAS format and archived with a CD backup. Statistical analyses were performed by using SPSS software for Windows (version 17.0; SPSS Inc., Chicago, Illinois). All incidences of the confirmed dengue were calculated as per 100 person-years (percent). Incidence rates in all the children and children aged ≤4, 5–9 and 10–14 years were determined by using the age-specific study population at the time of surveillance as denominator. Chi-square tests were used for determining the differences among the proportions of clinical spectra, dengue serotypes and annual incidences.

## Results

### Population characteristics

In February 2006, 3,015 students aged 3–13 years were enrolled in the study for the start of surveillance. In the subsequent 3 years, loss of students was a result of the graduation of sixth graders and families' relocation. Losses were 51 (1.7%) in 2006, 254 (8%) in 2007, and 384 (14%) in 2008. The higher dropout rate in 2008–2009 was due to 150 subjects' terminations. They were enrolled into Sanofi Pasteur's dengue vaccine efficacy trial which began in February 2009 [ClinicalTrials.gov Identifier: NCT00842530, http://www.clinicaltrials.gov]. Following replacement of dropouts, there were 3,220 (3–.14 years old) subjects in 2007 which declined to 2,833 (4–14 years) in 2008. Since there was no subject replacement in 2009, only 2,316 (5–15 years) subjects remained at the end of the study. No differences in gender distribution were noted from year to year or between schools (data not shown). Mean number of subjects enrolled in the four years was 2,846, with male to female ratio of 1.04∶1. Median age of participants shifted from 9 years in 2006 and 2007 to 10 years in 2008 and 11 years in 2009.

### Fever surveillance

Over the 4-year study period there were 36,934 student-absence episodes- 8,429, 9,438, 10,007 and 9,060, for each respective year of the study. During this same period there were 5,842 febrile illness episodes −1,892, 1,401, 1,527 and 1,022 for each respective year of the study. The mean student-absence episodes and mean febrile episodes per child per each study year are shown in table 1. Per year, the mean number of absences per student was 3.39 and each child had a mean febrile episodes of 0.53 (table 1).

**Table 1:** Incidence of laboratory confirmed symptomatic dengue cases, Na-muang subdistrict, Ratchaburi, Thailand, 2006–2009.

Year	No. students (person- year)	Mean number student absent (episode/child/year)	Mean number febrile illness (episode/child/year)	Incidence/100 person-year*
2006	2882.32	2.92	0.66	1.77
2007	3103.66	3.04	0.45	3.58
2008	2717.27	3.68	0.56	5.74
2009	2312.45	3.92	0.44	3.29

* There was statistically significant difference in the incidence of dengue year by year (p<0.001). Mean dengue incidence over the 4 years was 3.6%.

Of the study participants with a febrile illness over the 4-year study period, 73% were brought to RH by their parents. The majority of febrile children (53%) visited RH on days 1–2 after onset of fever, while 30%, 14% and 3% visited RH on days 3–4, days 5–6 and days 7–9 after onset of fever, respectively. In 2006, of 1892 children who had febrile illness, 734 (39%) came to RH of which 154 (8%) were admitted to the IPD and 580 (31%) required only OPD care. Twenty nine percent visited a private hospital and other clinics (IPD 1% and OPD 28%), and the remaining 32% of them bought medicine independently from pharmacy without seeking medical care (self treatment). The rate of hospital visits increased significantly from 39% in 2006 to 94% (1316/1401) in 2007, 88% (1347/1527) in 2008 and 87% (887/1022) in 2009. Numbers of febrile children who were admitted to RH IPD were 257 (18%) in 2007, 161 (10%) in 2008 and 90 (8%) in 2009.

### Dengue incidence

**Figure 2:**
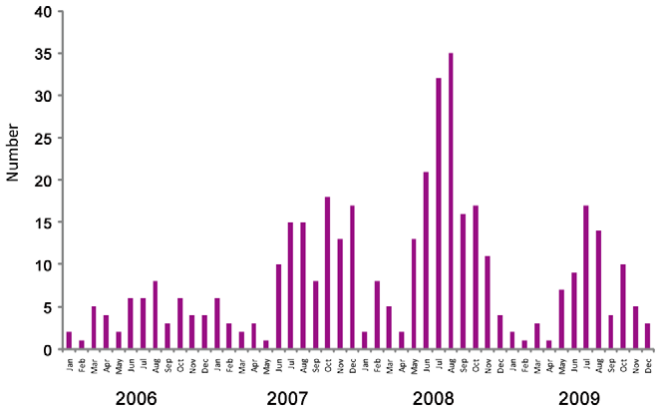
Number of serological confirmed dengue cases from January to December in 2006–2009.

During the study period 58.2% (3401of 5842) of febrile children had acute samples collected (S1: 625 from IPD and 2776 from OPD) and 57.7% (3368 febrile children) had paired sera collected (S2: 625 from IPD and 2743 from OPD) and tested for DENV and JEV infection. There were 394 serological confirmed dengue cases (215 boys and 179 girls). The case number was lowest (51 cases) in 2006, then rose to 111 cases in 2007, reached a peak at 156 cases in 2008 and declined to 76 cases in 2009. Dengue occurred all year round but the highest number was from June to August in 2006, 2008 and 2009. In 2007 the disease was highest from June to December (figure 2). Dengue accounted for 2.73%, 7.78%, 10.18% and 7.50% of the febrile illness episodes in each of the study years, respectively and averaged 6.74% of the children with a febrile illness.

**Figure 3:**
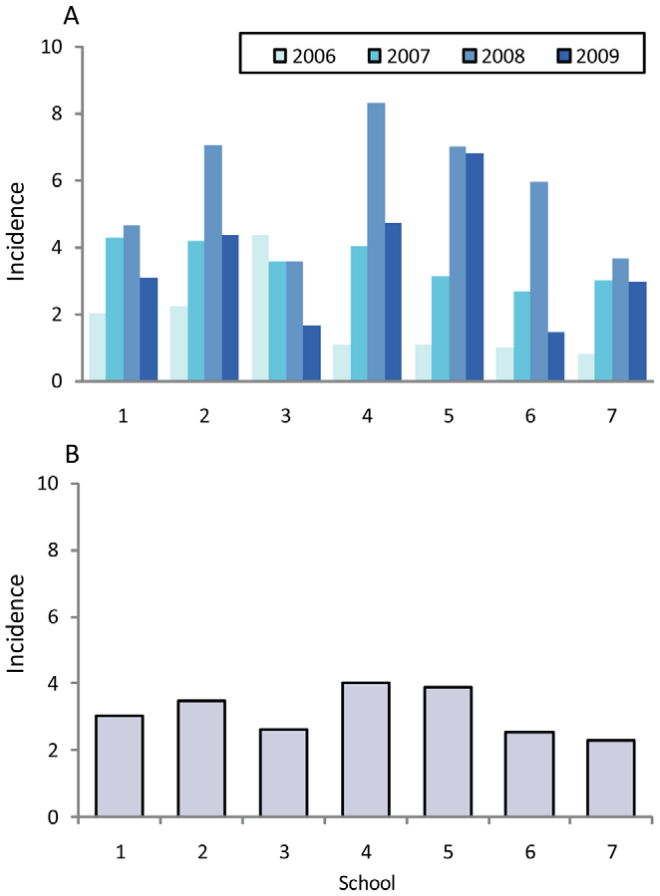
Dengue incidence. (**A**) Shown is the incidence by school in each year. (**B**) Shown is the incidence by school in all years.

Over the 4 years of the study, there were 2882.32, 3103.66, 2717.27 and 2312.45 person-years of follow-up for the respective years (table 1). There was statistically significant difference in the incidence of dengue year by year (p<0.001).The incidence in 2006 was the lowest (1.77%), increased to 3.58% in 2007, peaked in 2008 (5.74%) and then declined to 3.29% in 2009 (table 1). Mean dengue incidence over the 4 years was 3.6%. Dengue incidence varied by years and by schools. Over the 4 years, school 4 had the highest mean incidence (4%) and school 7 had the lowest mean incidence (2.31%) (figure 3). Among the 3,401 and 3368 samples of S1 and S2 tested, there were 9 serologically-determined acute JEV infection cases (6 and 3 cases in 2007 and 2008, respectively).

### Patient demographics, disease severity

Among the serologically confirmed 394 cases, 42 (10.7%) were DHF, 142 (36%) were DF and 210 (53.3%) were UF. Overall the proportion of males and females was 54.6% and 45.4%, respectively, and this male predominance persisted in all disease categories although it was not statistically significant (p = 0.89 chi square test). The age of cases ranged from 4–14 years (mean, 9.4; median, 10; mode 11) with slightly more cases in children aged 10–14 years old than in the 5–9 years old group: 200 (50.8%) vs. 192 (48.7%), respectively. The older age group also exhibited higher numbers of both DHF and DF (25 and 72 cases) than those of the younger age group (17 and 69 cases), respectively. However, there were no significant differences in disease severity, DHF vs DF between the two age groups (p>0.05, chi-square test). Only two children aged 3 and 4 years had dengue (one DF and one UF), so comparative analysis of the less than 4 year-age group with the older age groups was not performed. Mean ages for children with differing DHF grades was: I-11 years (n = 29), II- 10 years (n = 6) and III – 6 years (n = 7). Mean ages of DF and UF were 9.3 and 9.4 years, respectively. There were no deaths. Further details of clinical manifestations have been described [14].

### Dengue virus serotypes

Diagnosis of serotype was attempted on acute serum samples of 394 children who had serological confirmed dengue virus infection. These tests yielded DENV from 333 (84.5%) children, and the detection rate varied with only 67% in 2006 using mosquito isolation compared to 83% in 2007, 86% in 2008, and 96% in 2009 using RT-PCR. Viral detection also varied by day of sample collection, the highest rate (95%) was from the samples collected on days 1 and 2 after onset of fever, followed by days 3 and 4 (89%) and days 5 and 6 (69%).

All four DENV serotypes circulated in every study year (table 2). DENV-1 was the most common serotype detected with 144 isolates (43%) and predominated in every study year, ranging from 34 to 50 percent. DENV-2 (98 isolates, 29%) was the next most common, followed by DENV-3 (66 isolates, 20%) and DENV-4 (25 isolates, 8%). DENV-2 and DENV-3 had the lowest proportions of isolates in 2006 (3% and 9%, respectively). There was a 30 fold increase of DENV-2 to 33% of isolates in 2007 and a further increase to 37% of isolates in 2008 and declining to 25% of isolates in 2009. There was an 8 fold increase of DENV-3 to 26% of isolates in 2007, declining to 10% of isolates in 2008 and again rising to 34% of isolates in 2009. In 2006 DENV-4 was at its highest number: 13 isolates (38%), declining to 7, 3, and 2 isolates in 2007–9, respectively (table 2).

**Table 2:** Number and proportion of dengue virus serotypes (%) among dengue cases.

Year	DENV-1	DENV-2	DENV-3	DENV-4	UDS*	Total
2006	17( 50)	1(3)	3(9)	13(38)	17	**51**
2007	31(34)	30(33)	24(26)	7(8)	19	**111**
2008	68(51)	49(37)	14(10)	3(2)	22	**156**
2009	28(38)	18(25)	25(34)	2(4)	3	**76**
**All years**	**144 (43)**	**98 (29)**	**66 (20)**	**25 (8)**	**61**	**394**

* UDS, Unable to determine serotype, participants had IgM anti-DENV positive on S1 specimen or had seroconverted from IgM anti-DENV negative to positive between S1 and S2 specimens but dengue virus serotypes could not be identified from S1 [mosquitoes inoculation (2006) or RT-PCR (2007–9)].

**Figure 4:**
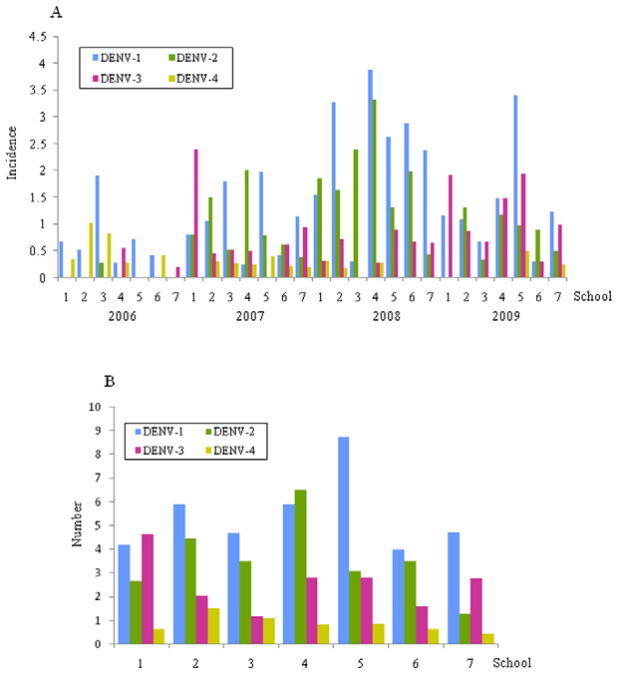
Serotype incidence. (**A**) Shown is the serotype incidence by school in each year. (**B**) Shown is the serotype incidence by school in all years.

DENV serotype specific incidence differed statistically by year (p<0.002) and varied between schools and between years (number of serotype-specific cases divided by number of children with acute dengue virus infection in the corresponding school). Over the study years, all four DENV serotypes were found in all 7 schools (figure 4). The highest incidence of DENV-1, DENV-2, DENV-3 and DENV-4 was found in schools 5, 4, 1 and 2, respectively.

### Disease severity, DENV serotypes, secondary DENV infection and hospitalization

All 4 DENV serotypes were associated with all illness categories (DHF, DF and UF) (table 3). DENV-1, DENV-2 and DENV-3 were associated with DHF grades 1, 2, and 3. DENV-4 was only observed among children with DHF grade 1.The proportions of DEN1, DEN2, DEN3 and DEN4 among children with DHF were 9.7%(14/144), 10.2% (10/98), 15.2% (10/66) and 8%(2/25), respectively. The proportions of DENV-1, DENV-2, DENV-3 and DENV-4 among children with DF were 39.6% (57/144), 31.6% (31/98), 39.4% (26/66), 44% (11/25), and in UF were 50.7% (73/144), 58.2% (57/98), 45.4% (30/66) and 48% (12/25), respectively. It was found that DENV serotypes had no significant correlation with disease severity (p>0.05 between DHF vs. non-DHF and p>0.05 between UF vs. non-UF) or for rate of hospitalization (p>0.05).

**Table 3:** Disease severity, virus serotype, secondary infection (2° infection), and hospitalization.

Clinical Diagnosis	DENV-1	DENV-2	DENV-3	DENV-4	UDS*
**DHF** (n, 42)	14	10	10	2	6
** *DHF Gr 1 (n, 29)*	*9*	*7*	*7*	*2*	*4*
*DHF Gr 2 (n, 6)*	*2*	*1*	*2*	*-*	*1*
*DHF Gr 3 (n, 7)*	*3*	*2*	*1*	*-*	*1*
2° infection (n, 40)	14	10	8	2	6
***Hospitalization (n, 42)	14	10	10	2	6
**DF** (n, 142)	57	31	26	11	17
2° infection (n, 126)	48	28	24	11	15
Hospitalization (n, 119)	52	23	22	9	13
**UF** (n, 210)	73	57	30	12	38
2° infection (172)	62	52	22	11	25
Hospitalization (32)	9	7	2	3	11

* UDS = Unable to determine serotype, participants had IgM anti-DENV positive on S1 specimen or had seroconverted from IgM anti-DENV negative to positive between S1 and S2 specimens but dengue virus serotypes could not be identified from S1 [mosquitoe inoculation ( 2006) or RT-PCR (2007–9)].

** DHF Grade 1, Grade 2, Grade 3.

*** 193 hospitalized patients consisted of DHF 42/42 cases (100%), DF 119/142 cases (83.8%), and UF 32/210 cases (15.2%).

Of all 394 dengue cases, secondary DENV infection was found in 86.3%, the remainder (13.7%) having a primary DENV infection (table 3). The rates of secondary DENV infection found in DHF, DF and UF cases were 95%, 88.7% and 81.91%, respectively. Primary DENV infection in 2 DHF Grade 1 cases was due to DEN3. There was no statistically significant difference in rate of secondary DENV infection in DHF (40/42) vs. those in DF and UF (298/352) (p>0.05).

Altogether 193 cases (47%) were hospitalized. The hospitalization rate by disease severity category was: DHF - 42(100%), DF - 119(84%), and UF - 32(15%). The proportion of hospitalized children with UF (32/172) was significantly lower than that of children with DF (119/142) (p<0.0001), which was significantly lower than those of DHF (42/42) (p<0.001) (table 3).

## Discussion

Our study is a population based epidemiological study driven by a clear objective of site preparation for a future dengue vaccine field trial. The Namuang sub-district of Muang district, Ratchaburi province, was chosen for the study site because it had high reported rates of dengue virus transmission. The incidence of dengue disease was determined from a prospective, long- term active fever surveillance of study participants in a well-defined cohort of school children throughout a 48-month period. Daily tracking of school absenteeism during school days and telephone contact with parents twice a week during school holidays made a broader capture of dengue possible. Most febrile illness cases detected in the cohort surveillance presented at Ratchaburi hospital were examined by pediatricians and had samples collected for dengue diagnostic testing.

An important aspect of this study was that our surveillance system captured children with febrile illness who sought medical care in both the public and private sectors. Prior to the beginning of the study, we met with a private hospital director and pediatricians who worked on the private hospital and clinics in Muang district to inform them of the study project. They were asked to inform us whenever participants attended their hospital/clinics. As a result, our staff visited all participants admitted to the private hospital and obtained acute serum samples from febrile patients. Convalescent samples were subsequently collected at RH. In addition, by end of the first year, we had strengthened parent-staff communications and education by use of telephone explanation and written documents that explained provision of study care with no charge fast track service and telephone consultation service at RH. Our efforts to educate parents were evidenced by increased in parent initiated hospital visits for febrile illness to 87% in the years 2007–2009.

Our rates of dengue incidences over the four-year period, 1.77, 3.58, 5.74 and 3.29 per 100 person-years in 2006–2009 reflect a parallel pattern of dengue incidence reported from the national surveillance data base in 2006–9 for Muang district, Ratchaburi Province: 159, 200, 361 and 155 per 100,000, respectively. Our prospectively determined dengue incidence in Na-muang sub-district is 11 to 21 (average 16.5) fold higher than those derived from the national surveillance database in Muang district. One difference between our incidence rates and the national rates is that we prospectively determined DHF, DF and UF cases among febrile illnesses of primary school children, whereas, the national data presents mainly DHF reported from hospitalized patients in all age groups.

Although there were some methodological differences in how active surveillance was conducted in our study and previously reported studies in childhood cohorts from Kampang Phet, Thailand [15], Kampong Cham, Cambodia [16] and Managua, Nicaragua [17], the incidences of laboratory confirmed dengue found by active surveillance for acute febrile illness were much higher than that of the corresponding national reporting data. Using a calculated expansion factor to estimate differences between national reporting and laboratory based surveillance for dengue incidence, Wichmann et al. 2011 found the average under-recognition of dengue across three cohort studies (Kampang Phet, Ratchaburi and Kampong Cham) of dengue incidence to be more than 8-fold [18]. The Nicaraguan study found under-reporting of dengue cases in relation to national surveillance systems to be 21.3 fold [17]. The national under-reporting of dengue incidence of cases hinders accurate knowledge of disease burden.

All four DENV serotypes were involved in all levels of disease severity observed over the study period. Although there was strong seasonal variation, laboratory confirmed dengue virus infections were observed in every month of the four-year observation period in all 7 schools indicating that there is continuous DENV transmission in Ratchaburi. DENV-1 was the most commonly virus isolated (43% of total), followed by DENV-2 (29%) and DENV-3 (20%). New introduction in 2007 of DENV-2 and DENV-3 after a period of relatively low frequency in occurrence was followed by a severe outbreak in 2008 wherein DEN2 frequency was 30-fold increased and DENV-3 8-fold increased. This observation is consistent with a previous report that in Thailand DENV-2 was a dominant isolate during moderately severe dengue outbreak years and DENV-3 was associated with subsequent severe outbreaks after a time of relatively low frequency [6].

Dengue virus isolation methods differed between 2006 and the subsequent years in that PCR was used from 2007–2009. A lower DENV isolation rate was observed when culture in C6/36 cells and mosquito inoculation (67%) were used compared to detection of virus genome using RT-PCR (>83%) in this study is likely due to lower sensitivity of mosquito methods compared to PCR molecular methods [19], [20]. The overall viral identification rate using combined mosquito inoculation and PCR tests of 84.5% reported in this study is higher than the 65% in the previous report which also used both methods [7].

Secondary-type DENV infection described in this study was based on the ratio of anti-DENV IgM: IgG<1∶8 [11]. It has been observed from serology test results that there is cross reaction between JEV IgG and DENV IgG in S1 as well as in S2 with resulting inability to distinguish JEV IgG from DENV IgG. This observation had also been reported previously [21]. As the Thai National Immunization Program has included JE vaccination since 2000, JEV IgG from vaccination might have had some influence on the observed DENV IgG titers and may have confounded the interpretation of secondary infection data reported here. However, the majority (84.5%) of the patients with secondary DENV infection was accompanied with positive dengue virus detection and had low JEV IgG titer in their acute (S1) specimens suggesting a minimal impact on the validity of anti-DENV IgM/IgG ratio used to estimate secondary DENV infection.

There appears to have been a shift in modal age of dengue incidence over the past four decades in Thailand. A retrospective hospital based study of laboratory confirmed dengue in 15,376 patients from Bangkok, Thailand reported a modal age of 5 years during 1973–79 which increased to 8 years during 1990–99 [6]. We found a modal age of 11 years in the present study. This finding might reflect a somewhat older group presenting with less severe disease (UF) in outpatient settings, but it is consistent with the increasing modal age previously reported [6]. Hospitalization rates for acute symptomatic dengue infection could be considered a measurement of dengue disease severity. The hospitalization rate for DHF, DF, and UF were 100- 84- and 15- percent, respectively. Over the four years of our study, the provision of fast track service without payment, led to progressively better compliance of febrile cases from the cohort reporting directly to hospital OPD for evaluation, minimizing the possibility that cases may have been missed, especially in years 2007–9. However it is possible that the study missed some mild febrile cases that did not attend RH in 2006.

Because of the sensitivity of our surveillance system only 184 (46%) of laboratory confirmed dengue cases (DHF 42 cases and DF 142 cases) met the 1997 WHO case definition [1]. Clinical manifestations of 210 (52.5%) of laboratory confirmed UF cases did not meet the WHO case definition for dengue [1]. UF was the most common clinical manifestation of children infected with dengue virus, whether primary or secondary DENV infections, and can be difficult to distinguish from other childhood febrile illnesses [14]. Our findings that a high proportion of dengue cases manifested as UF has an important impact on present vector control practices. In Ratchaburi province, mosquito control campaigns against dengue occur broadly over the province, four times per year. In addition, implementation of mosquito control measures including source reduction, application of larvicides, and spraying residual insecticide, are implemented in the area of 100 meters in diameter around all the reported houses of patients with dengue infection at all times of year. Due to the current national surveillance system only DHF/DF cases observed in clinical facilities are reported. Mosquito control measures are not implemented around houses of UF patients. This may contribute to inability to adequately control dengue and result in the long persistence and wide spread of dengue in the province as well as in Thailand in general.

We performed an extensive dengue epidemiology study and report accurate background data on dengue incidence in a cohort of individuals at high risk of dengue, children ages 3–15 years living in Namuang subdistrict of Muang district of Ratchaburi province, Thailand. We found sufficiently high incidence of four DENV serotypes for four consecutive years to make vaccine efficacy studies possible. Our study methods and findings fulfilled the epidemiological criteria recommended by WHO for dengue vaccine trial site selection [8]. Following the reports of safety and immunogenicity of phase I studies of live-attenuated tetravalent dengue vaccine in dengue–naïve and dengue-endemic populations [22], [23], the site was selected for the first dengue vaccine efficacy and safety trial, phase 2b. This trial uses a live-attenuated yellow fever vaccine virus based chimeric tetravalent dengue vaccine in a 3-dose vaccine administration of doses spaced at six-month intervals. This trial was launched in early 2009 and dose administration in approximately 4000 participants was completed in March 2011 [ClinicalTrials.gov Identifier: NCT00842530). Safety of the vaccine within the trial enrollment and vaccine administration period has been established. An analysis for vaccine efficacy is planned in 2012. If the vaccine is sufficiently immunogenic and efficacious after long term follow up, it is anticipated that the vaccine might be available for use in 2015.

## Supporting Information

Checklist S1 **STROBE statement.** Checklist of items is included in this cohort study.
(DOC)
